# Alteration of Innate Immunity by Donor IL-6 Deficiency in a Presensitized Heart Transplant Model

**DOI:** 10.1371/journal.pone.0077559

**Published:** 2013-10-16

**Authors:** Fangmin Ge, Shunzong Yuan, Lida Su, Zhonghua Shen, Aibin He, Tao Huang, Weihua Gong

**Affiliations:** 1 Department of Surgery and Medicine, Transplant International Research Centre (TIRC), Second Affiliated Hospital of School of Medicine, Zhejiang University, Hangzhou City, People's Republic of China; 2 Department of Lymphoma, Affiliated Hospital of Academy of Military Medical Sciences, Beijing, People's Republic of China; 3 Department of Medicine, Beth Israel Deaconess Medical Center, Harvard Medical School, Boston, Massachusetts, United States of America; 4 Department of Cardiology, Children's Hospital Boston, Harvard Medical School, Boston, Massachusetts, United States of America; Beth Israel Deaconess Medical Center, Harvard Medical School, United States of America

## Abstract

Engraftment of IL-6 deficient donor into wild-type recipient could significantly improve allograft survival through T cell lineage particularly regulatory T cells (Tregs) in non-sensitized transplant host. However, its effect on innate immune responses remains uncertain. Our data revealed that donor IL-6 deficiency significantly increased infiltration of two subsets of MDSCs (CD11b+Gr1+myeloid-derived suppressor cells), CD11b+Gr1^-low^ and CD11b+Gr1^-int^ with strong immunosuppression activity in the transplanted graft. It resulted in a dramatic increase of CD11b+Gr1^-low^ frequency and a significant decrease of the frequency of CD11b+Gr1^-high^ and CD4-CD8-NK1.1+ cells in the recipient’s spleen. Unexpectedly, donor IL-6 deficiency could not significantly reduce macrophage frequency irrespective of in the host’s spleen or graft. Taken together, suppression of innate immune effector cells and enhanced activity of regulatory MDSCs contributed to tolerance induction by blockade of IL-6 signaling pathway. The unveiled novel mechanism of targeting IL-6 might shed light on clinical therapeutic application in preventing accelerated allograft rejection for those pre-sensitized transplant recipients.

## Introduction

Interleukin-6 (IL-6) as a pleiotropic cytokine can be produced by various tissues particularly proinflammatory cell types and formed in local cytokine milieu. It is capable of orchestrating cellular differentiation, affecting early graft function recovery [1], and disrupting transplant tolerance induction [2,3]. IL-6 produced from injured allograft vessels could promote the magnitude of intimal expansion and allogeneic T cell infiltration through suppressing an increase of CD161+ regulatory T cells [4]. The multifaceted activities of IL-6 can be characterized by inhibition of Th1 response, impairment of suppressive function of regulatory T cells (Tregs), and promotion of Th2 and Th17 lineage development, directing against transplant tissue [5-7]. IL-6 amplifier and NF-kB-triggered positive feedback for IL-6 mediate in vitro hyperinduction of chemokine ligand 2 (CCL2) by IFN-γ in type 1 collagen cells, contributing to allogeneic responses and graft rejection [8].Therefore, it was observed that donor graft-derived but not recipient source IL-6 becomes a systemic danger signal impairing constitutive immune suppression. Accumulating experimental and clinical evidences revealed that intragraft IL-6 gene expression level is closely associated with chronic [9] and acute graft rejection [3,7]. Elevation of urinary IL-6 concentration and serum IL-6R (IL-6 receptor) level was reported to be a suitable biomarker for predicting an early acute transplant rejection episode [10]. Conversely, donor IL-6 deficiency prolongs allograft survival via the presence of regulatory CD25+ T cells [3]. Neutralization of IL-6 can reduce T cell infiltration and decrease Th17 markers [4], rescuing early graft function [1] and significantly prolonging allograft survival [2,3,7]. 

Indeed, not only adaptive but innate immune cells are required for acute or chronic rejection in various transplant settings [11]. A recent study reported that TLR4/TRIF pathway contributes to allogeneic bone marrow cell rejection dependent on innate immune cells including F4/80+ (macrophages) or NK1.1+ cells (NK cells), causing a significant production of proinflammatory cytokine IL-6 and TRIF relevant chemokine MCP-1 [12]. During transplantation surgery the mechanical and ischemia-reperfusion injuries are inevitable, which causes microbial productions or endogenous pro-inflammatory ligands [11]. Thereafter, necrotic lysates released from graft can trigger higher inflammatory responses in dendritic cells (DCs) and natural killer cells, resulting in a remarkable production of inflammatory mediators including TNF-α and IL-6 in a MyD88-dependent manner, an insusceptibility of alloreactive T cells to suppression by CD4^+^CD25^+^ regulatory T cells (Tregs), and an initiation of T-cell immunoresponses against graft [2,11]. By contrast, absence of both IL-6 and TNF-α can result in permanent acceptance of allograft [2]. 

Although impact of IL-6 on adaptive immune responses was described in different transplant models [2,3,7], its specific contribution to the process of innate immune responses remains to be elucidated. To this end, we utilized a pre-sensitized transplant model for studying the effect of IL-6 on innate immune responses. A deeper understanding of the role of IL-6 during transplantation may pave the way to a more success in identifying novel transplant tolerance induction strategies [11].

## Materials and Methods

### Animals

Eight-twelve week male inbred mice were obtained from the Jackson Laboratories (JAX, Bar Harbor, ME) including wild-type C57BL/6 (B6; H-2^b^; Stock No. 000664), BALB/c (B/c; H-2^d^; Stock No. 000651), and B6:129S2-IL-6^tm1Kopf^/J (IL-6KO; H-2^b^; Stock No. 002650). The mice were housed under pathogen-free conditions at the Center for Life Science (Boston, MA). Animal studies were approved by the Institutional Animal Care and Use Committee (IACUC) at Beth Israel Deaconess Medical Center. Allogeneic heterotopic heart transplantations (HTx) were performed using BALB/c donors and C57BL/6 recipients.

### Vascularized heterotopic heart transplantation

Abdominal heterotopic heart transplantation was performed using standard microsurgical techniques as previously described [13,14]. Briefly, the mice were anesthetized with an intraperitoneal injection of a mixture of ketamine (60 mg/kg) and xylazine (10 mg/kg). The pulmonary artery and the ascending aorta of donor were anastomosed end-to-side to the recipient’s inferior vena cava (IVC) and the abdominal aorta. Graft function was daily monitored by observation of donor heartbeat palpation. The happening of rejection was judged when heart beating was completely ceased and later confirmed by histology. The cardiac grafts that did not function at day 1 post-transplantation were not included. 

**Figure 1:**
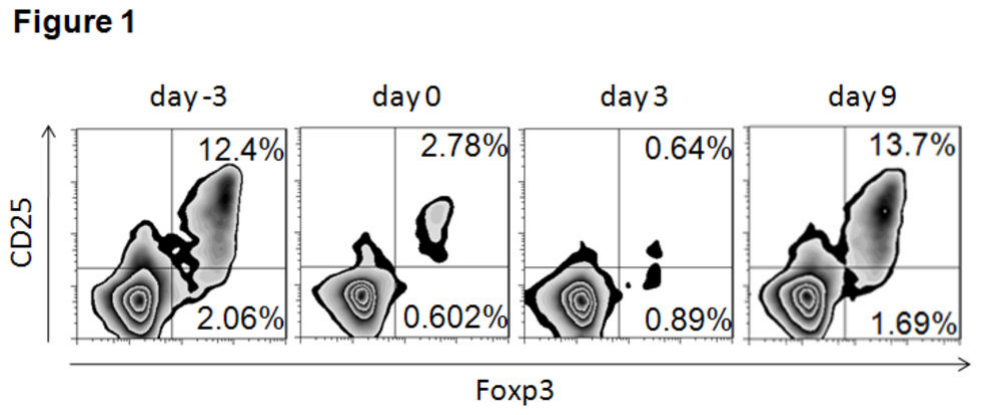
Administration of anti-CD25 mAb (PC61) depleted CD4+CD25+Foxp3+ cells (n=2). Peripheral blood CD4+CD25+Foxp3+ T cells was analyzed through flow cytometry at day -3 (pre-administration of anti-CD25 monoclonal antibody), day 0 (transplant surgery), day 3, and day 9 post-transplant surgery. Administration of anti-CD25mAb 3 days before transplant surgery resulted in a continuous decrease of frequency of peripheral CD4+CD25+Foxp3+ T cells (12.4% on day -3, 2.78% on day 0, 0.64% on day 3). On day 9, the frequency of peripheral CD4+CD25+Foxp3+ T cells reactively increased owing to acute rejection event.

Experimental groups are shown in Table 1. Briefly, to test the role of donor IL-6 cytokine in the non-sensitized transplant settings, IL-6 deficient (knockout with C57BL/6 background) mice were used as donor and Balb/c mice acted as recipients (G1). These recipients were treated an isotype control iso-IgG as controls. To prove involvement of Tregs for the protective role of donor IL-6 deficiency, anti-CD25 monoclonal depleting antibodies (mAb) (0.25 mg, clone PC61, BD Pharmingen) were intraperitoneally applied 3 days before transplantation for aforementioned transplant recipients (G2). Tregs depletion was confirmed by an analysis of peripheral CD4+CD25+ cells frequency at day -3 (pre-administration of anti-CD25 mAb), day 0 (transplant surgery), day 3, and day 9 post-transplant via flow cytometry (Figure 1). In addition, allogeneic (G3) and syngeneic (G4) transplantations were performed as control groups. These four groups (G1-G4) were established to generate allograft survival curves. Furthermore, to investigate the impact of donor IL-6 deficiency on innate immune system, PCI-mediated injury in the pre-sensitized transplant settings was utilized for our study based on our previous researches [15]. C57BL/6 recipients pre-sensitized by Balb/c skins at transplant day -7 were engrafted with wild-type C57BL/6 hearts with PCI (7.0±0.5 hours, G5) or IL-6 deficient (IL-6KO) C57BL/6 hearts with PCI (6.5±0.8 hours, G6). This PCI time can partially cause cardiac graft injury rather than heart-beat failure (unpublished data). 

**Table 1:** Displays design of experimental groups of non- or pre-sensitization and the treatment characteristics.

**Group**	**n**	**Skin for sensitization**	**Donor for HTx**	**PC61 (0.25mg, i.v.)**	**PCI (hrs**)
G1	7(5) ^ξ^	No	IL-6KO	No	No
G2	9(5) ^ξ^	No	IL-6KO	Yes	No
G3	7(5) ^ξ^	No	C57BL/6	No	No
G4	6(6) ^ξ^	No	BALB/c	No	No
G5	3	BALB/c	C57BL/6	No	7.0±0.5
G6	4	BALB/c	IL-6KO	No	6.5±0.8

ξ The number in the parenthesis represents amount of transplant surgeries for survival curves among total surgeries (n).

### Reagents and antibodies

#### Immunofluorescence analysis and mAbs

Harvested spleen and heart cells were directly labeled with fluorescently conjugated monoclonal antibodies (mAbs). All mAbs used for cell surface staining were purchased from BioLegend (San Diego, CA). For FACS staining, anti–mouse CD4 (Clone H129.19, Catalogue No. 130308) FITC-conjugated mAb, anti–mouse CD8a (Clone 53–6.7, Catalogue No. 100701) PE-conjugated mAb, Alexa Fluor® 700-conjugated anti-mouse Gr-1/Ly-6G (Clone RB6-8C5, Catalogue No. 108421) mAb, and the pacific blue-conjugated anti–mouse CD11b (clone M1/70, Catalogue No. 101223) mAb were used to label live T cells. All samples were acquired using an LSRII (BD Biosciences, Mountain View, CA). Data was analyzed using FlowJo 7.5 software (Tree Star, Ashland, OR), as we reported before [16]. The analysis gate was set on the side and forward scatters to eliminate debris and dead cells cell.

### Isolation of graft-infiltrating leukocytes

The heart grafts were harvested, minced and then incubated in Roswell Park Memorial Institute (RPMI) 1640 medium containing 0.5 mg/mL collagenase IV (Sigma-Aldrich, St. Louis, MO, USA) for 30 min at 37°C in the shaker. Tissue debris was carefully removed with 70-µm cell strainer. After washing twice, viable mononuclear cells were separated by Ficoll density gradient centrifugation. The cells at the interface were isolated and washed thrice with RPMI 1640 medium supplemented with 10% FBS and then subjected to either fluorescence-activated cell sorting (FACS) staining.

### Histology and Immunohistochemical Analysis

Cardiac grafts were harvested at day 9 post-transplantation. Heart specimens were fixed in 10% paraformaldehyde and then embedded in paraffin. Tissue was sectioned at 5µm-thickness. These sections were stained with hematoxylin and eosin (H&E) and assessed by light microscopy to determine the extent of cellular infiltration.

Frozen heart graft specimen were sectioned at 5-μm-thickness and immunohistochemistry was performed using an avidin-biotin-peroxidase technique (Vector Laboratories, Inc, Burlingame, CA), with ImmPACT DAB Chromogen. Heart sections were blocked with goat serum and then labeled with an anti- Mouse Ly-6G (Gr-1, Clone IA8, Catalogue No. 551495), a monoclonal antibody (eBiosience, San Diego, CA), which recognizes neutrophils. Specimen sections were incubated using the Avidin-Biotin Complex Solution, Vectastain Elite ABC kit (Vector Laboratories, Inc, Burlingame, CA). ImmPACT DAB Chromogen was applied and subsequently counter-stained with Harris hematoxylin. 

### Statistical analysis

Allograft survival curves were generated by using the Kaplan-Meier method. All data were analyzed using the statistical software GraphPad Prism version 5.0 (GraphPad Software, San Diego, CA). The FACS data between groups were determined by a student T-test. The difference of graft survival between different groups was compared by both log-rank (Mantel-Cox Test) and Gehan-Breslow-Wilcoxon Test. A p-value of .05 was determined as statistical significance. The FACS data were expressed as mean ± SD.

## Results

### Prolonged Survival of IL-6 Deficient Heart Allografts

**Figure 2:**
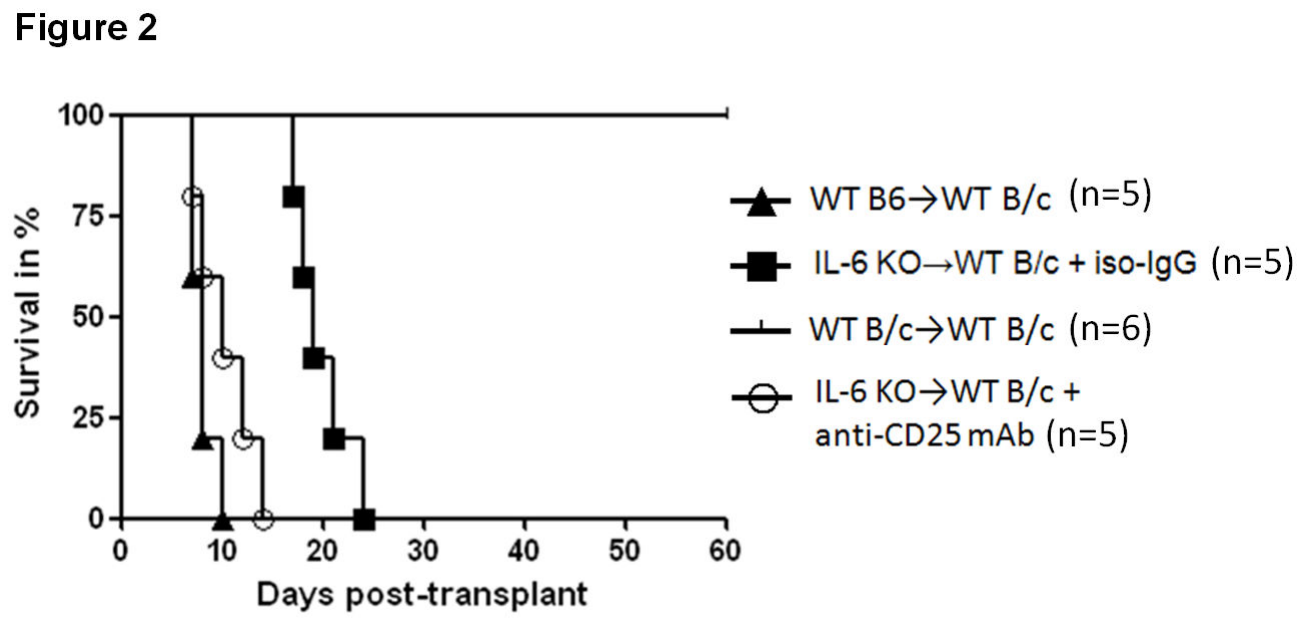
Kaplan-Meier cardiac graft survival curve. Heterotopic abdominal heart transplantation was performed by using standard procedure. All transplanted mice were monitored every day until graft rejection, defined as the cessation of palpable cardiac activity. Although only three symbols of “triangle” are observed for group (WT B6B/c), two grafts survived for 7 days and two grafts survived for 8 days. It implies that each “triangle” on day 7 (60% remained to survive on day 7) and day 8 (20% remained to survive on day 8) represents two grafts. Graft survival of the allogeneic control group (C57BL/6BALB/c, n=5) is equivalent to IL-6 deficiency donor graft (IL-6KOBALB/c) treated with anti-CD25 mAb (n=5) (Mantel-Cox Test, p=0.17; Gehan-Breslow-Wilcoxon Test, p=0.28), whereas IL-6 deficiency donor graft survival (IL-6KOBALB/c) treated with iso-IgG (n=5) was significantly prolonged (Mantel-Cox Test, p=0.012; Gehan-Breslow-Wilcoxon Test, p=0.0031) in comparison to allogeneic control group.

Previously, various experimental studies demonstrated a critical role of donor IL-6, which mainly originated from the donor allograft tissue and could impair allograft survival [3]. This data was supported by our present studies, in which utilization of IL-6 deficient donor caused a prolonged allograft survival. The mean survival time (MST) of IL-6-/- heart grafts in the wild-type BALB/c recipients (IL-6KO B/c) was significantly increased (19.8±2.8 days) compared to that of allogeneic grafts (B6 B/c) (MST=8.0±1.2 days; Mantel-Cox Test, p=0.0012; Gehan-Breslow-Wilcoxon Test, p=0.0031) (Figure 2).

**Figure 3:**
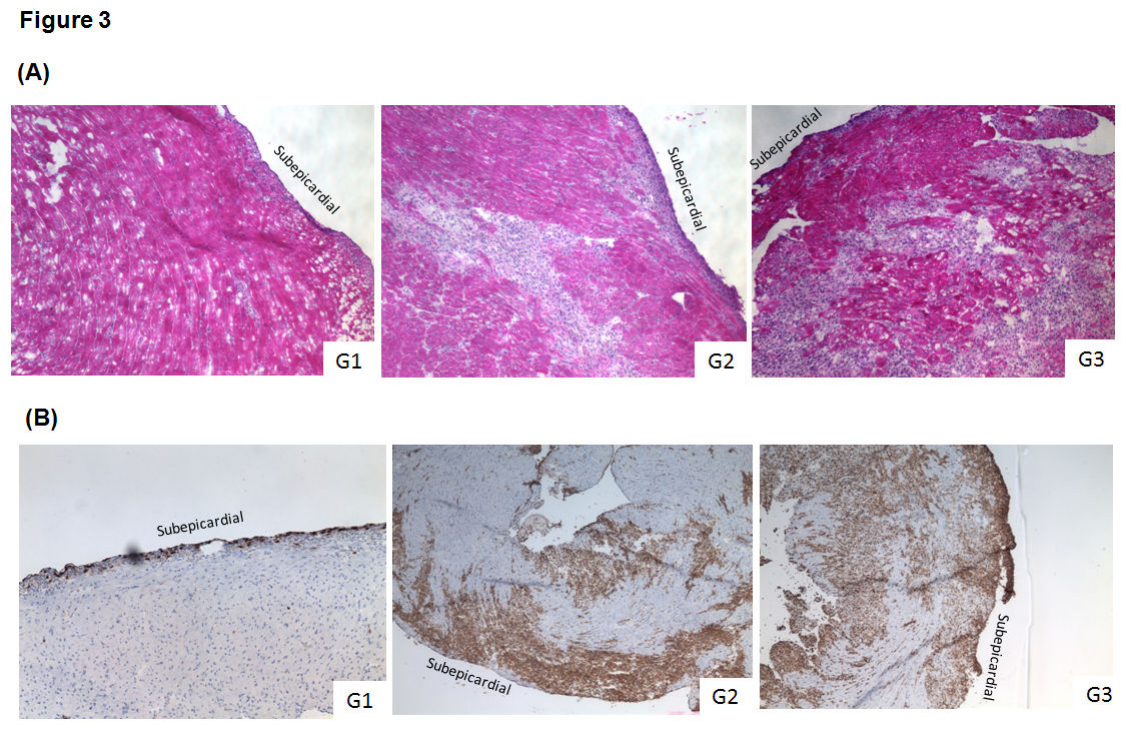
Graft histology and immunohistochemical analysis of grafts. (A) The heart transplanted recipients were sacrificed at day 9 after surgery. Allogeneic grafts were harvested, stained with H&E to assess inflammation and lymphocytes infiltration between different groups under microscope, as described in the Methods section. The figure shows that a mild leukocytes infiltration was observed in the IL-6 deficient donors (G1) (n=2), while a pronouncedly infiltration of leukocytes and preserved cardiac architecture were observed in the subepicardial area of the IL-6KO grafts treated by anti-CD25 mAb (PC61) (G2) (n=2) and wild-type allografts (G3) (n=2) without any treatment. (B) Grafts specimens harvested at day 9 post-transplant were snap-frozen into Tissue Tec and stained for Gr-1 protein as described in the Materials and Methods section. Immunohistochemical sections showed that depletion of Treg cells using anti-CD25 mAb (PC61) remarkably caused an infiltration of Gr-1+ cells in the IL-6 deficient grafts, which is similar to acutely rejected wild-type hearts. A mild subepicardial Gr-1+ cells infiltration was found within IL-6KO grafts without any treatment. Original magnification ╳ 40.

However, additional use of anti-CD25 mAb (PC61) to those IL-6-/- heart grafts (IL-6-/-BALB/c) pronouncedly shortened graft survival equivalent to allogeneic control group (B6 BALB/c) (Figure 2). An increase of frequency of peripheral CD4+CD25+Foxp3+ regulatory T cells (Figure 1) was caused owing to acute rejection event, accounting for a protective reactivity of recipient in the settings of strong effect of effector cells. It demonstrated that depletion of Tregs resulted in the poor transplant outcome despite lack of donor IL-6 cytokine, indicating that regulatory T cells were involved in the protective effect of donor IL-6 deficiency. Afterwards, pathological and immunohistochemical analysis were performed to further validate this findings. As a result, mild subepicardiac graft infiltrating leukocytes (GILs) particularly reduction of Gr1+ neutrophils were observed, while donor IL-6 deficiency combined with Tregs depletion caused a severe infiltration of leukocytes and Gr1+ neutrophils and cardiac structure destruction, accounting for acute rejection events on day 9 post-transplantation (Figure 3A and 3B). 

### Characterization of innate immune cells in PCI-mediated injury in the pre-sensitized transplant host engrafted with IL-6 deficient donor

**Figure 4:**
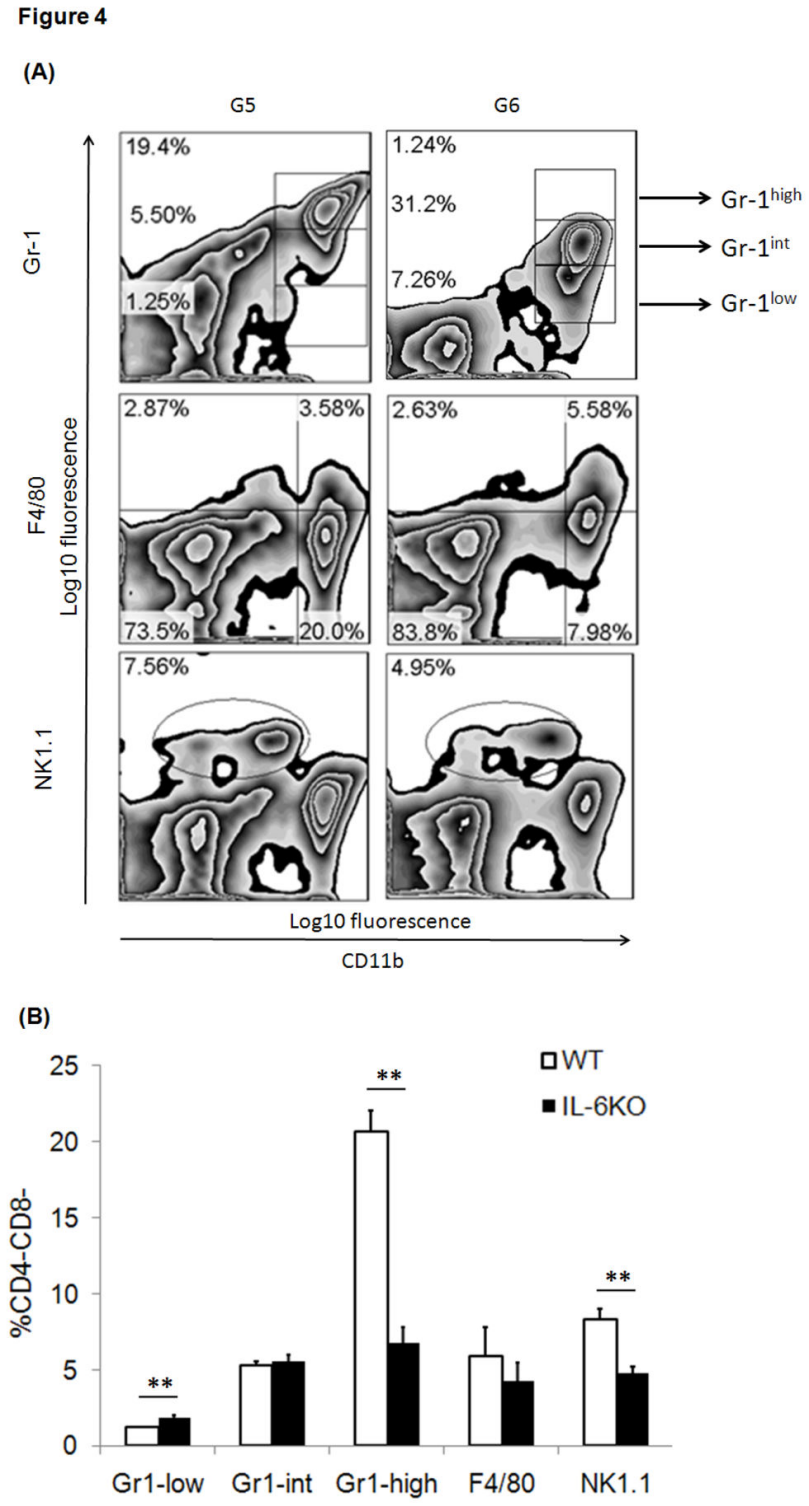
(A) The frequency of MDSCs, CD4-CD8-NK1.1+, F4/80+ cells in the spleen of the transplant recipient. These innate immune cells in the spleens were collected for fluorochrome-labeling and subject to FACS analysis on day 3 after transplant. (B) Statistical analysis was performed for the groups G5 (n=3) and G6 (n=4). *p<0.05, **p<0.01.

To clearly identify the impact of PCI on allograft in the sensitized recipients, we dissected the process of accelerated transplantation and utilized syngeneic cardiac transplantation with PCI after recipients were pre-sensitized by fully MHC-mismatched skin. Based on the evident effect of IL-6 abrogation in the non-sensitized transplant model, we attempted to test whether lack of intragraft IL-6 cytokine could cause any significant alteration of innate immune response in the pre-sensitized transplant host’s spleen. Based on aforementioned data on the critical role of Tregs in the transplantation, we firstly tested non-specific innate immunoregulatoy cells, myeloid-derived suppressor cells (MDSCs), which have been thoroughly studied in our previous research. MDSCs can be sub-grouped into three different subsets, CD11b+Gr1^-low^, CD11b+Gr1^-int^, and CD11b+Gr1^-high^ with distinct immunoregulatory effect. Therefore, we tried to characterize MDSCs subsets, macrophage, NK1.1+ cells in the spleen of wild-type donor + PCI (G5) and IL-6 deficient donor + PCI (G6) by flow cytometry analysis (Table 1). As a result, our studies revealed that donor IL-6 deficiency caused mild reduction of macrophage (F4/80+) frequency (G5 vs. G6: p=0.3), a dramatic increase of CD11b+Gr1^-low^ frequency (p=0.0021) and a significant decrease of CD11b+Gr1^-high^ (p=0.00015) and CD4-CD8-NK1.1+ cells (p=0.0017) frequency in the recipient’s spleen (Figure 4). 

#### Significant accumulation of CD11b+Gr1^-low^ and CD11b+Gr1^-int^ MDSCs within IL-6 deficient graft

**Figure 5:**
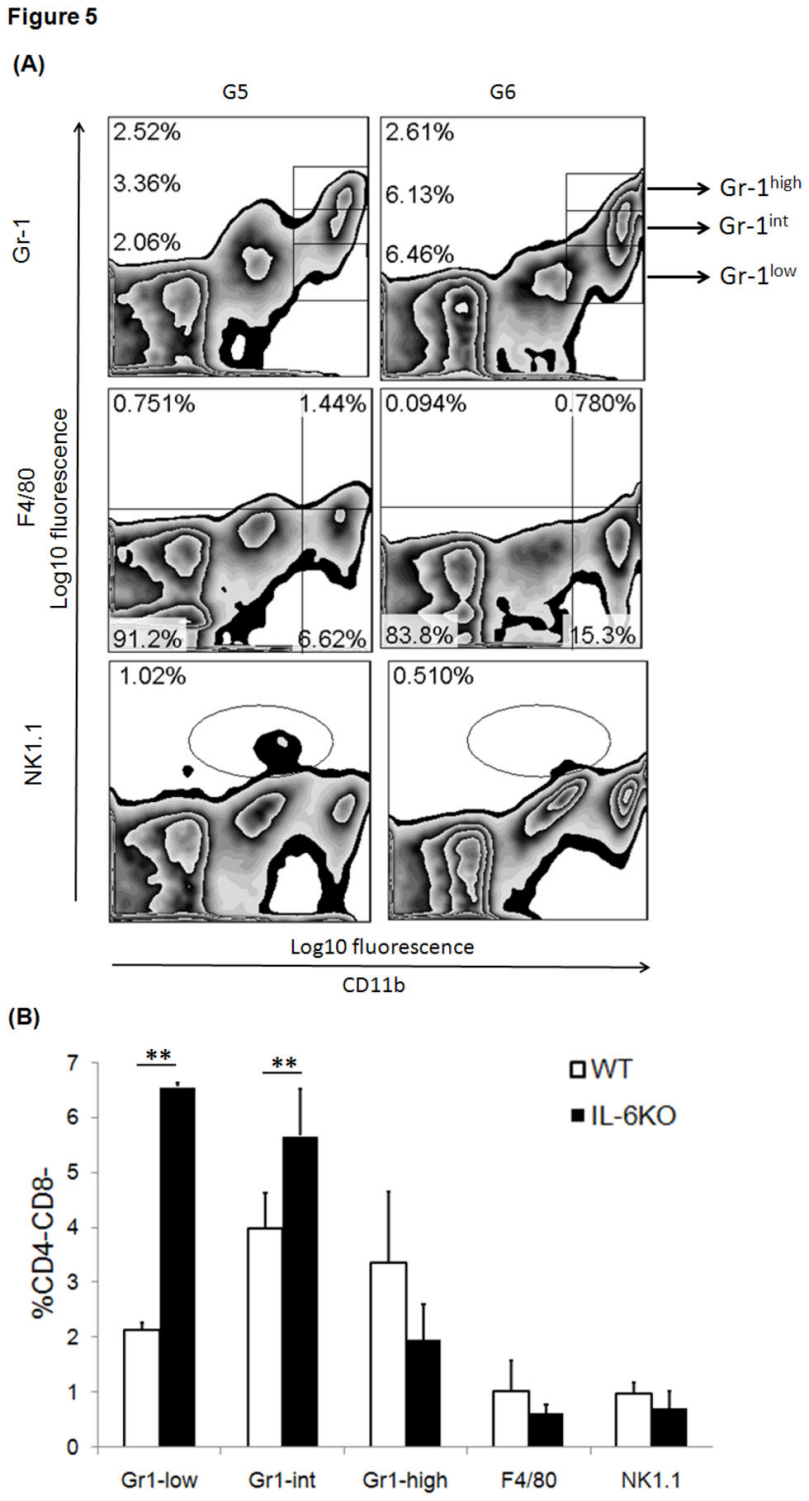
(A) The frequency of MDSCs, CD4-CD8-NK1.1+, F4/80+ cells within cardiac graft of the transplant recipient. Graft-infiltrating these innate immune cells were collected for fluorochrome-labeling and subject to FACS analysis on day 3 after transplant. (B) Statistical analysis was performed for the groups G5 (n=3) and G6 (n=4). *p<0.05, **p<0.01.

To detect whether donor IL-6 deficiency affects two important subsets of MDSCs, CD11b+Gr1^-low^ and CD11b+Gr1^-int^ with strong immunosuppression activities, the pre-sensitized transplanted grafts with PCI including IL-6-deficiency and wild-type were harvested. The infiltrated leukocytes within graft were isolated and subject to cytometry analysis. The findings revealed that donor IL-6 deficiency did not remarkably suppress infiltration of macrophage within graft (p=0.3), while it caused a significant increase of intragraft CD11b+Gr1^-low^ (p=0.00001) and CD11b+Gr1^-int^ frequencies (p=0.032) in the early stage (Figure 5). 

## Discussion

The presensitized PCI-mediated transplant model was selected for two reasons. First, prior to empirical transplant surgery a considerable amount of transplant recipients have undergone previous failed transplants, multiple blood transfusions, ventricular assist device placement, or pregnancies, more likely to endanger hosts to alloantigen-sensitization situation. Our previous novel findings revealed that pre-sensitized immune condition of recipient can exaggerate PCI-mediated injury of heart grafts. Second, owing to scarcity transplant organ much effort has been made to utilize remote donors, leading to a prolongation of cold-ischemic time during organ transportation.

Allogeneic immune responses directing allograft are the main obstacle to transplant tolerance induction [7]. Past studies on graft acceptance and survival mainly focus on adaptive immune cells including B and T cells, and their long-lasting immunological memory cells [17]. The fact that IL-6 contributes to numerous adaptive immunologic effects associated with transplant rejection has been fully described [1-3,7-9]. Herein, we attempted to appreciate the critical role of donor IL-6 deficiency in innate alloimmunity under pre-sensitized PCI-mediated injury condition.

Numerous cell types accounting for alloimmune responses can culminate in heart graft rejection [7]. Evidence has shown that allogeneic immune responses are characterized by monocytes/macrophages and neutrophils infiltration within grafts. Monocyte is able to infiltrate allograft within 20 hours, rapidly differentiate into mature dendritic cells (DCs), and elicit immunoresponse as donor and recipient differs at non-MHC loci. In lung transplantation monocyte prior to neutrophil migrates to graft in a CCR2-dependent manner [18,19]. Adoptive transfer of monocytes from alloimmunized host can confer allorecognition to naïve host [18]. Furthermore, it was found that at day 3 posttransplantation a vast majority of intragraft DCs are derived from recipient’s monocytes [18]. With respect to NK cell, it can express both activating and inhibitory receptors despite the missing-self theory, lack of self-MHC molecules on cell surface [18]. Activated NK cell can kill target cells via lysis and then bear memory property. In addition, NK cell is capable of shaping adaptive immune responses through producing cytokines, which orchestrate B and T cell response [20]. Studies have shown that NK cells are necessary and sufficient for host to chronically reject allograft [21,22]. Nevertheless, depletion of NK cells in wildtype mice cannot effectively attenuate acute rejection even in combination with T cells depletion [22]. Dr. Lakkis FG et al. revealed that NK cells are capable of downregulating the homeostatic proliferation of CD8+ T cells under lymphopenic conditions, delaying allograft rejection [23]. Therefore, it was proposed that NK cells do not possess a critical link between innate and adaptive immune responses under transplant condition [18]. 

Therefore, our present study tested the role of those innate immune cells including macrophage, NK1.1+ cell, and MDSCs subsets in the setting of PCI-mediated injury in the pre-sensitized transplant host. The findings manifested that donor IL-6 deficiency mounted a weaker immune response in such pre-sensitized transplant recipient. At day 3 post-transplantation macrophage frequency in the graft (Figure 4B) and spleen (Figure 5B) remained decreased despite no significance. In addition, donor IL-6 deficiency resulted in a significant decrease of CD4-CD8-NK1.1+ cells frequency in the recipient’s spleen. MDSCs featured as innate immunoregulatory cells, are able to inhibit allogeneic T cells proliferation and allorecognition (submitted to *Cell Transplantation*, manuscript No. CT-1098). Thereafter, it is reasonable to explain that remarkable infiltration of two main subsets of MDSCs, CD11b+Gr1^-low^ and CD11b+Gr1^-int^ was observed within donor IL-6 deficient graft. A dramatic increase of CD11b+Gr1^-low^ frequency and a significant decrease of CD11b+Gr1^-high^ frequency were found in the recipient’s spleen. 

By far, it is well-studied that heart is the main source producing cytokine IL-6 [3]. The inflammatory process of transplantation followed by ischemia/reperfusion can cause disturbance of mitochondria, and DNA escaping from flamed heart [24]. Any mitochondrial DNA escaping the degradation might bind to TLR9, capable of inducing cardiomyocytes to produce IL-1β and IL-6 [25]. In a setting of CD8+ cell-dominant rejection process, blockade of IL-6 could delay the onset of acute rejection, prevent graft infiltration and reverse the ratio of Th1/Th2 in host, while in the setting of CD4+ cell-dominant graft rejection, blockade of IL-6 could significantly prolong graft survival associated with reduced graft infiltration, altered Th1 responses, and inhibited production of serum alloantibody [7]. Nevertheless, the specific pathway of IL-6 toward triggering allogeneic innate immunoreponses remains unclear particularly the role of regulatory innate immune cells, MDSCs and further investigations are required to clarify the details of underlying molecular mechanisms.

Taken together, our present study revealed that blockade of IL-6 signaling pathway suppressed innate immune effector cells in large measure and enhanced activity of regulatory MDSCs. This unveiled novel mechanism of targeting IL-6 might shed light on clinical therapeutic application in preventing accelerated allograft rejection for those pre-sensitized transplant recipients.
